# Comparative Loss-of-Function Screens Reveal ABCE1 as an Essential Cellular Host Factor for Efficient Translation of *Paramyxoviridae* and *Pneumoviridae*

**DOI:** 10.1128/mBio.00826-19

**Published:** 2019-05-14

**Authors:** Danielle E. Anderson, Kristin Pfeffermann, So Young Kim, Bevan Sawatsky, James Pearson, Mikhail Kovtun, David L. Corcoran, Yvonne Krebs, Kristmundur Sigmundsson, Sharon F. Jamison, Zhen Zhen Joanna Yeo, Linda J. Rennick, Lin-Fa Wang, Pierre J. Talbot, W. Paul Duprex, Mariano A. Garcia-Blanco, Veronika von Messling

**Affiliations:** a Programme in Emerging Infectious Diseases, Duke-NUS Medical School, Singapore, Singapore; b Veterinary Medicine Division, Paul-Ehrlich-Institute, Langen, Germany; c Department of Molecular Genetics and Microbiology, Duke University, Durham, North Carolina, USA; d Duke Center for RNA Biology, Duke University, Durham, North Carolina, USA; e Programme in Cardiovascular and Metabolic Disorders, Duke-NUS Medical School, Singapore, Singapore; f National Emerging Infectious Diseases Laboratories, Boston University, Boston, Massachusetts, USA; g INRS-Institut Armand-Frappier, University of Quebec, Laval, Canada; h Department of Biochemistry and Molecular Biology, University of Texas Medical Branch, Galveston, Texas, USA; Johns Hopkins Bloomberg School of Public Health; St. Jude Children's Research Hospital; University of California, Irvine

**Keywords:** ABCE1, *Paramyxoviridae*, *Pneumoviridae*, RNAi screen, host factor, respiratory syncytial virus

## Abstract

Paramyxoviruses and pneumoviruses have similar life cycles and share the respiratory tract as a point of entry. In comparative genome-scale siRNA screens with wild-type-derived measles, mumps, and respiratory syncytial viruses in A549 cells, a human lung adenocarcinoma cell line, we identified vesicular transport, RNA processing pathways, and translation as the top pathways required by all three viruses. As the top hit in the translation pathway, ABCE1, a member of the ATP-binding cassette transporters, was chosen for further study. We found that ABCE1 supports replication of all three viruses, confirming its importance for viruses of both families. More detailed characterization revealed that ABCE1 is specifically required for efficient viral but not general cellular protein synthesis, indicating that paramyxoviral and pneumoviral mRNAs exploit specific translation mechanisms. In addition to providing a novel overview of cellular proteins and pathways that impact these important pathogens, this study highlights the role of ABCE1 as a host factor required for efficient paramyxovirus and pneumovirus translation.

## Abstract (precis)

The *Paramyxoviridae* and *Pneumoviridae* families include important human and animal pathogens. To identify common host factors, we performed genome-scale siRNA screens with wild-type-derived measles, mumps, and respiratory syncytial viruses in the same cell line. A comparative bioinformatics analysis yielded different members of the coatomer complex I, translation factors ABCE1 and eIF3A, and several RNA binding proteins as cellular proteins with proviral activity for all three viruses. A more detailed characterization of ABCE1 revealed its essential role for viral protein synthesis. Taken together, these data sets provide new insight into the interactions between paramyxoviruses and pneumoviruses and host cell proteins and constitute a starting point for the development of broadly effective antivirals.

## INTRODUCTION

Members of the family *Paramyxoviridae* have recently been reclassified into two families: *Paramyxoviridae* and *Pneumoviridae* (1). Both families include a broad range of respiratory viruses with great relevance for human and animal health. These viruses all have nonsegmented RNA genomes of negative polarity, and many of the viral proteins share considerable structural and functional homology within a genus and even between genera (2, 3). The negative-strand RNA genome by itself is not infectious. Instead, the viruses rely on their own incoming polymerase complex for transcription and replication (4, 5), resulting in relative independence from cellular processes during these infection stages. Thus, cellular proteins involved in different aspects of the *Paramyxoviridae* and *Pneumoviridae* life cycle are much less well characterized than those of other virus families.

The availability of genome-scale loss-of-function screens has enabled an unbiased investigation of the role of cellular proteins in diverse processes (6, 7). As obligate intracellular parasites, viruses critically depend on the exploitation of the cellular environment for their amplification, and genome-scale siRNA screens of infected cells have yielded new insights in the host cell functional interactome of many virus families (8, 9). For negative-strand RNA viruses, several proteins within the coatomer complex I (COPI), cellular proteins essential for the secretory pathway, were originally identified in a vesicular stomatitis virus (VSV) genome-scale siRNA screen (10) and then found to also be required for replication of human parainfluenza virus 3 (HPIV3) (10), suggesting a role for this pathway in the life cycle of several negative-strand RNA viruses. Screens with human respiratory syncytial virus (HRSV) revealed actin-related protein 2 and the calcium pump SPCA1 as host factors involved in different aspects of particle maturation, egress, and spread, the last of which is also required for other RNA viruses (11, 12). In addition, the large ribosomal subunit protein 40, one of the hits in the VSV screen, has been shown to be specifically needed for the translation of negative-stranded RNA virus mRNAs (13), but other host factors involved in viral mRNA translation remain to be identified.

Measles virus (MeV), mumps virus (MuV), and HRSV are representatives of the *Paramyxoviridae* and *Pneumoviridae* families with high clinical relevance. All three viruses are transmitted by aerosol or large droplets as well as through contact and fomites. MeV and MuV result in systemic infection, whereas HRSV remains restricted to the respiratory tract (2). While the MeV and MuV vaccines are among the most successful vaccines available today (14–18), an approved vaccine for HRSV remains an unmet need. Aside from a prophylactic monoclonal antibody-based treatment against HRSV, no approved therapeutics are available (19). Given the general propensity of these viruses to infect the respiratory tract, identification of common cellular host factors could lead to rapid symptom-based treatment options with rationally designed broad-spectrum therapeutics. Towards this end, and to learn more about the biology of these viruses, we performed comparative genome-scale siRNA screens of the three viruses. To reproduce the common natural environment as closely as possible, the human lung adenocarcinoma cell line A549, which shares many key features with the respiratory epithelial cells (20), was selected for the screens. In addition to the previously identified secretory pathway (10), our screens identified translation, proteasomal degradation, and RNA processing as important host cell pathways for the life cycles of all three viruses.

After secondary screening of candidates and comparison with a screen for Hendra host factors, we chose to focus our studies on the ATP-binding cassette (ABC) transporter ABCE1. ABCE1 is a member of the superfamily of ABC transporters that contain two nucleotide-binding domains and two N-terminal iron-sulfur clusters. Unlike most ABC domain proteins, ABCE1 lacks a membrane-spanning domain and therefore is unlikely to have a transporter function (21). ABCE1 plays an important role in translation (22), as it is crucial for ribosome recycling *in vivo* and controls ribosome homeostasis (23, 24). Additional roles have also been attributed to this protein (25–27). ABCE1 was initially identified as an inhibitor of endoribonuclease L (RNase L) (28). More recently, ABCE1 was demonstrated to act as an endogenous suppressor of RNA silencing (29), and during HIV infection, it plays a critical role in particle formation by associating with assembly-competent HIV Gag polypeptides (30–32). Here we show that ABCE1 knockdown strongly inhibits the translation of MeV mRNAs while only modestly affecting global cellular translation, suggesting that these mRNAs, and likely mRNAs from all *Paramyxoviridae* and *Pneumoviridae*, may be particularly dependent on the function of this protein.

## RESULTS

### Genome-scale RNAi screens for host factors involved in the *Paramyxoviridae* and *Pneumoviridae* life cycles.

**FIG 1:**
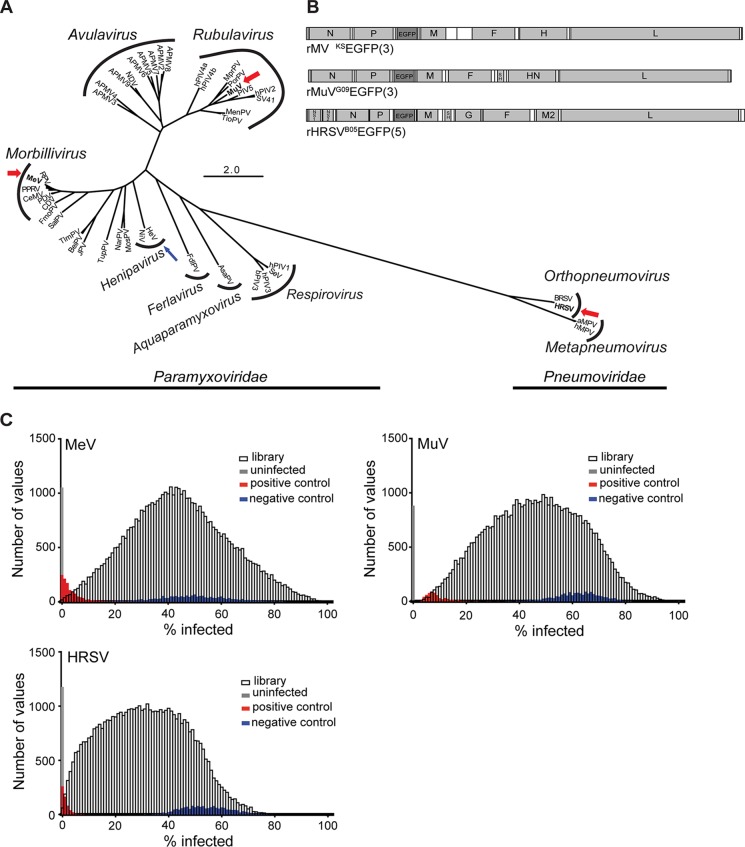
Selection of viruses and RNAi screen performance. (A) A total of 45 N protein sequences were downloaded from GenBank: measles virus (MeV), rinderpest virus (RPV), canine distemper virus (CDV), pestes-des-petits ruminants virus (PPRV), phocine distemper virus (PDV), cetacean morbillivirus virus (CeMV), mumps virus (MuV), human parainfluenza viruses 1 to 4 (hPIV1 to -4), porcine rubulavirus (PorPV), Mapuera virus (MprPV), parainfluenza virus 5 (PIV5), Menangle virus (MenPV), Tioman virus (TioPV), Newcastle disease virus (NDV) avian paramyxoviruses 2 to 9 (APMV 2 to -9), Sendai virus (SeV), bovine parainfluenza virus 3 (bPIV3), Hendra virus (HeV), Nipah virus (NiV), J paramyxovirus (JPV), Beilong paramyxovirus (BeiPV), Salem virus (SalPV), Tupaia paramyxovirus (TupPV), Nariva virus (NarPV), Mossman virus (MosPV), fer-de-lance virus (FdlPV), Atlantic salmon paramyxovirus (AsaPV), bovine respiratory syncytial virus (BRSV), human respiratory syncytial virus (HRSV), avian metapneumovirus (aMPV), and human metapneumovirus (hMPV). Amino acids were aligned using the MUSCLE plugin in Geneious 7.1.6. Alignments were visually inspected and manually curated. The best protein evolution model was determined according to Akaike information criterion (AIC) scores using ProtTest. An LG+G+F maximum likelihood analysis was run in PHYML using average likelihood ratio test (aLRT) statistics for branch support. MeV, MuV, and HRSV are boldfaced and highlighted with a thick red arrow to indicate that the viruses were screened, and HeV is boldfaced and highlighted with a thin blue arrow to indicate that data from a separate screen (43) were included in candidate host factor selection. (B) Schematic drawing of recombinant viruses. Gray and white boxes represent open reading frames and UTRs, respectively. The genes are indicated by their respective abbreviations. The inserted EGFP gene is shaded in dark gray, and the number in parentheses indicates the position of the gene in the viral genome. (C) Histogram of percent infection distribution for MeV, MuV, and HRSV. Distribution of percent infection values for each of the individual virus screens. For reference, distribution of positive (red), negative (blue), and uninfected (gray) controls is shown for each screen.

To identify host proteins important for *Paramyxoviridae* and *Pneumoviridae* pathogenic to humans, independent genome-scale siRNA screens were conducted with MeV and MuV, human pathogens of the *Morbillivirus* and *Rubulavirus* genera, respectively, within the *Paramyxoviridae* family, and HRSV of the genus *Orthopneumovirus* within the *Pneumoviridae* family (Fig. 1A). Recombinant EGFP-expressing derivatives of wild-type MeV, MuV, and HRSV strains (Fig. 1B) were used to facilitate quantification of infected cells by detection of green fluorescence (33–35). To ensure comparable results, all screens were performed in the lung adenocarcinoma cell line A549 engineered to stably express the human MeV receptor SLAM (A549-hSLAM).

We used a Qiagen genome siRNA library designed to target 21,705 human genes, with at least four siRNAs per gene, formatted in two pools of two siRNAs each (see Fig. S1A in the supplemental material). Target annotation for the library was updated using the GRCh37 human assembly, resulting in a small minority of genes being targeted with more or less than two pools. At 48 h after siRNA transfection, cells were infected with the respective virus at an MOI determined to lead to infection of approximately 50% of cells. When infected cells could be clearly identified and the cytopathic effect was still limited, which corresponded to 33 h postinfection (hpi) for the faster-growing MeV, and 72 hpi for MuV and HRSV, the percentage of infected cells was analyzed using a Cellomics ArrayScan system (Fig. S1B). Infection levels in the majority of negative-control wells ranged from 45 to 65% and revealed idiosyncratic behavior for each virus, which is not surprising for individual siRNAs (Fig. 1C). Importantly, positive-control siRNAs against EGFP or COPA dramatically reduced the percentage of EGFP-positive cells for all viruses (Fig. 1C, red bars) nearly to the level of uninfected cells (gray bars). For MeV and HRSV, the distribution of the siRNA-treated wells was shifted toward lower infection levels, whereas the distribution in the MuV screen was almost normal (Fig. 1C). For MeV and MuV, the distribution of the siRNA-treated wells relative to that of the controls enabled the reliable identification of hits on both ends of the phenotypic spectrum, while for HRSV, the behavior of the negative controls suggested potentially less reliable identification of antiviral factors (see Table S1).

10.1128/mBio.00826-19.1 FIG S1 Schematic overview of the RNAi screens and representative images of infection levels in individual wells. (A) Individual genome-wide screens for MeV, MuV, and RSV were performed as indicated. All screens were individually analyzed to determine robust Z scores, and then the three screens were compared. (B) Identification of infected cells. Following infection with either MeV, MuV, or RSV, cells were fixed and stained with Hoechst 33342. Stained cells were imaged and analyzed with a Cellomics ArrayScan VTI automated microscope. All images shown are captured from wells transfected with nonsilencing control siRNA. Panels on the top show infected cells, as indicated by EGFP expression. Panels on the bottom show quantification of infected cells. Uninfected cells served as a reference population for background fluorescence. Images were collected by autofocusing on nuclear staining in channel 1. Cells were then identified in channel 1, indicated as valid object count (highlighted in orange). Percentage of cells infected with EGFP-expressing virus was determined by analyzing cells for presence of EGFP signal in channel 2 (highlighted in blue). Download FIG S1, PDF file, 2.9 MB. Copyright © 2019 Anderson et al. 2019 Anderson et al. This content is distributed under the terms of the Creative Commons Attribution 4.0 International license.

10.1128/mBio.00826-19.4 TABLE S1 Antiviral host factor ranked list of genes with *P* and *q* values for combined analysis, based on analysis of the raw data using informatics tools that do not rely on the behavior of the negative controls (86). Download Table S1, XLSX file, 0.7 MB. Copyright © 2019 Anderson et al. 2019 Anderson et al. This content is distributed under the terms of the Creative Commons Attribution 4.0 International license.

**FIG 2:**
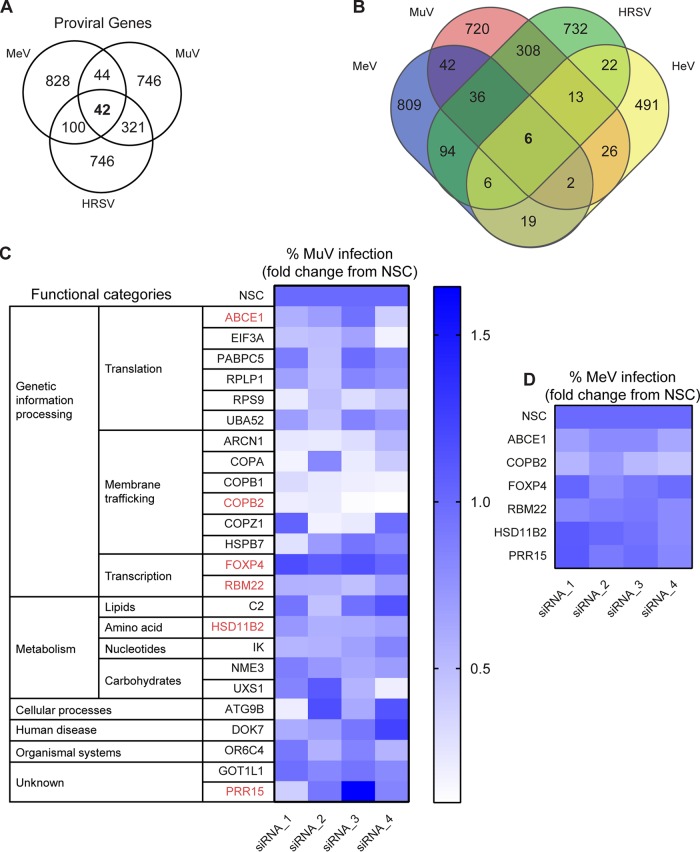
Identification and validation of common proviral hits. (A) Common *Paramyxoviridae* and *Pneumoviridae* host factors. Extent of overlap among the genes required for the replication of MeV, MuV, and RSV. The number of genes inhibiting the replication of the viruses in both A/B and C/D siRNA sets (robust Z score < 0.8) is tabulated in the Venn diagram. (B) Extent of overlap among the genes required for the replication of MeV, MuV, RSV, and HeV. The number of genes inhibiting the replication of the viruses is tabulated in the Venn diagram. Multiple siRNAs were used to target each of the six top-ranking genes identified by comparative analysis of the three primary screens and the previously published HeV screen (43). (C) MuV validation screen of 24 common hits identified by both Z score and KS analysis. Forty-eight hours after siRNA transfection, cells were infected with MuV at a multiplicity of infection of 1.0 and incubated for 48 h. The percentage of infected cells was calculated, and the mean percent infection was set at 1 for the NSC. The heat map shows the fold change compared to the NSC, and each square represents the average from three experiments for each individual siRNA. Functional categorization of genes is shown beside the heat map. The gene hits shown in red were then selected for MeV validation screening. (D) MeV validation screen of top proviral hits also identified in the HeV screen. Forty-eight hours after siRNA transfection, cells were infected with MeV at a multiplicity of infection of 0.05 and incubated for 48 h. The percentage of infected cells was calculated, and the mean percent infection was set at 1 for the NSC. The heat map shows the fold change compared to the NSC, and each square represents the average from five experiments for each individual siRNA.

The identification of the COPI complex, which is known to be required for negative-strand RNA viruses (10), as a proviral factor for all three viruses (Fig. 2A) and the MeV receptor SLAMF1 for only the MeV screen (Table S2) provided confidence that the screening and analysis approaches were appropriately chosen to identify novel paramyxovirus host factors. The complete data sets for all three screens are provided in Table S2.

10.1128/mBio.00826-19.5 TABLE S2 Complete MeV, MuV, and RSV genome-scale siRNA data set. Raw data and robust Z scores for cell number and percent infection for each screen. Download Table S2, XLSX file, 5.9 MB. Copyright © 2019 Anderson et al. 2019 Anderson et al. This content is distributed under the terms of the Creative Commons Attribution 4.0 International license.

### Analysis of individual screens reveals unique host factors for each virus.

Each of the genome-scale screens identified candidate proviral factors uniquely required for MeV, MuV, or HRSV or shared by only two of these viruses (Fig. 2A). Interestingly, MeV and MuV each shared more dependency factors with HRSV than they did with each other, which is surprising given their phylogenetic relationship. Proteins involved in the cellular translation machinery and control of the cell cycle and, surprisingly, the activation of the innate immune response were also prominently represented among the top proviral factors in each of the individual screens (Table S2). For MeV, synaptotagmin 8, which is expressed in islet cells and involved in insulin expression (36), and insulin itself were among the top hits, suggesting a hitherto-unknown role of glucose metabolism in MeV infection. Furthermore, the calcium/calmodulin-dependent protein kinase type II beta chain (CAMK2B), which was previously identified as a host factor supporting influenza virus RNA transcription (37), also supports MeV replication. The proapoptotic serine/threonine kinase 3 (STK3), one of the top hits in the MuV screen, also acts as a proviral factor for influenza virus (38).

Actin-related protein 2, identified as a proviral host factor involved in virus egress and dissemination in a similar screen using the tissue-culture adapted HRSV A2 strain (12, 39), did not reach significance in our screen. However, cofilin 1, which associates with the viral matrix (M) protein in HRSV particles and may modulate particle assembly and release (40, 41), was among the top hits. Furthermore, dynamin 2 was also identified as an important HRSV host factor. Dynamins are part of the endocytosis pathway and were recently implicated in HRSV entry in airway epithelial cells (42). Thus, our data also provide insights into the unique requirements for these viruses or their genera.

### Comparative meta-analysis identifies common candidate proviral factors.

The screens were first analyzed separately using a custom bioinformatics package that calculates statistical significance for genes and pathways by considering the distribution of all wells that belong to a specific gene or pathway by Kolmogorov-Smirnov (KS) testing. As a further step, we performed a meta-analysis of the three screens that yielded 179 genes required for all three viruses (false discovery rate < 0.05), which are thus candidate host proviral factors for *Paramyxoviridae* and *Pneumoviridae* (highlighted in Table S3). The list of candidate proviral factors was significantly enriched for gene products involved in RNA processing, mRNA translation, and proteasome-mediated degradation (Table S4).

10.1128/mBio.00826-19.6 TABLE S3 Host factors identified by KS analysis. One hundred seventy-nine host factor genes are highlighted in green. Download Table S3, XLSX file, 0.7 MB. Copyright © 2019 Anderson et al. 2019 Anderson et al. This content is distributed under the terms of the Creative Commons Attribution 4.0 International license.

10.1128/mBio.00826-19.7 TABLE S4 Proviral host factor ranked list of pathways with *P* and *q* values for combined analysis. Download Table S4, XLSX file, 0.03 MB. Copyright © 2019 Anderson et al. 2019 Anderson et al. This content is distributed under the terms of the Creative Commons Attribution 4.0 International license.

To narrow down the list of the most promising pan-paramyxovirus host factor genes, we performed a complementary meta-analysis of the three data sets using robust Z score-based metrics. We identified putative host factors as genes with at least two independent siRNA pools with Z scores of −0.8 or below across all three screens (Table S2). This second and very different analysis approach resulted in the identification of only 42 proviral gene products required by all three viruses (Fig. 2A). Of these, 24 hits were also identified by the initial KS analysis (Table S5), and we consider these 24 to be the highest-confidence list of candidate proviral factors broadly required by *Paramyxoviridae* and *Pneumoviridae*. Six of these hits (ABCE1, COPB2, FOXP4, HSD11B2, PRR15, and RBM22) were also identified in a screen for Hendra virus (HeV) host factors in HeLa cells using a different siRNA library (43) (Fig. 2B), and one (UXS1) was identified in an HRSV screen in human haploid cells (11). It must be clear that these common hits, which we address here, were not necessarily among the top hits for each individual screen. To examine the lists of most potent proviral hits for each individual screen, we refer the reader to Table S2.

10.1128/mBio.00826-19.8 TABLE S5 Proviral host factor genes identified by Z score analysis in MeV, MuV, and RSV screens. Download Table S5, PDF file, 0.2 MB. Copyright © 2019 Anderson et al. 2019 Anderson et al. This content is distributed under the terms of the Creative Commons Attribution 4.0 International license.

### Validation screens confirm membrane trafficking and translation as proviral pathways.

To assess the importance of these common proviral proteins in the *Paramyxoviridae* life cycle, we next performed a validation screen of the 24 candidates identified by both meta-analysis approaches using MuV, since an almost normal distribution of infection in the siRNA-treated wells was observed in the initial siRNA screen with this virus (Fig. 1C). Consistent with the fact that most of the 24 common hits were not among the strongest hits for each of the individual screens, the overall extent of inhibition of infection was moderate (Fig. 2C). A complementary validation screen with MeV of the six genes that were also among the proviral factors identified in the HeV screen (Fig. 2B) yielded similar results (Fig. 2D).

Among the top common proviral genes identified in our analysis were six components of the coatomer complex I (ARCN1, COPA, COPG1, COPB1, COPB2, and COPZ1), and pathway enrichment analysis highlighted multiple vesicle- and COPI-mediated transport pathways (Tables S2, S4, and S5). The COPI complex is involved in retrograde vesicular transport from the Golgi complex to the endoplasmic reticulum (44) and was previously reported to be involved in the life cycle of several negative-stranded RNA virus families, including paramyxoviruses (10, 43, 45). Our data also revealed important roles for components of the translation machinery: ABCE1, EIF3A, and RPLP1 (Fig. 2C and D and Tables S2, S4, and S5). While it was previously understood that general translation components would be absolutely required, our analysis highlights specific requirements for *Paramyxoviridae* and *Pneumoviridae*. For instance, we noted a disproportionate dependence on proteins of the small ribosomal subunit (e.g., RPS9, which appears in Fig. 2C and Table S5), something that was not observed in similar screens for flaviviruses (46). Of interest is also the requirement for the ribosomal protein RPLP1, and the likely requirement of its partner RPLP2, which did not satisfy the most stringent cutoff criteria but was among the 179 gene products identified as common hits in the KS analysis. These two components of the ancient ribosomal stalk, which are dispensable for bulk cellular translation, are exquisitely required for flavivirus translation (46, 47), suggesting a widespread role in the translation of viral proteins.

### ABCE1 supports replication of MeV, MuV, and HRSV.

**FIG 3:**
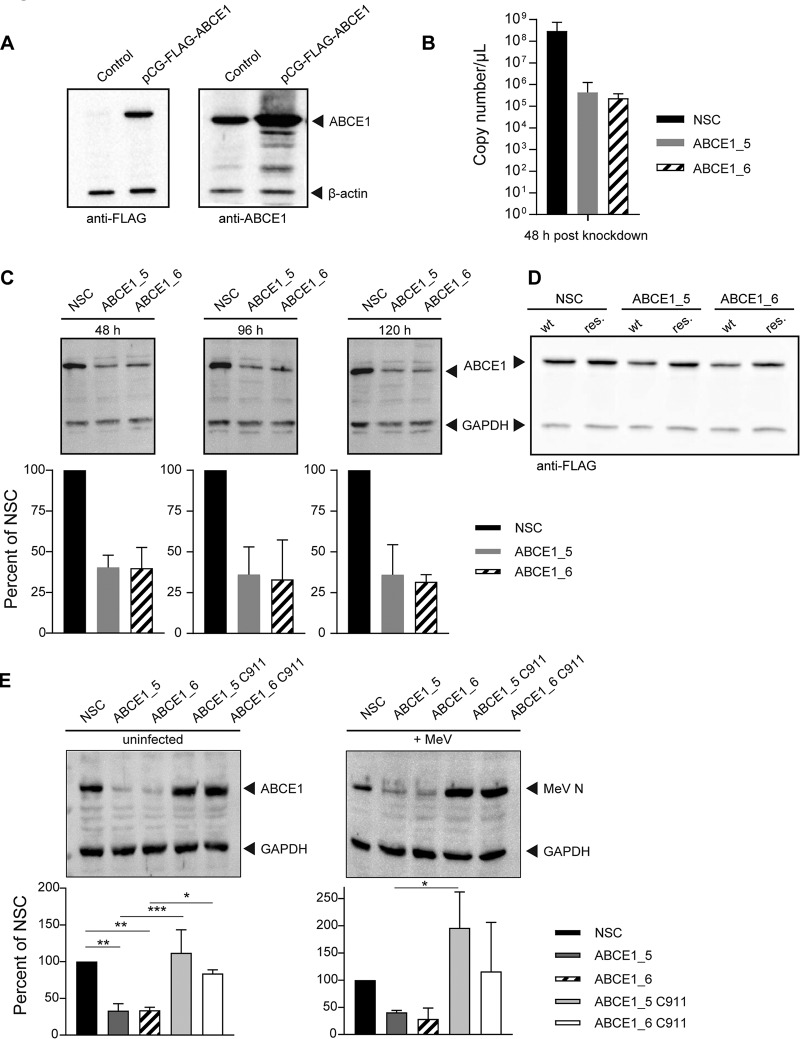
ABCE1 protein expression levels and knockdown efficiency. (A) Confirmation of rabbit anti-ABCE1 peptide antiserum specificity. A549-hSLAM cells were transfected with an expression plasmid coding for human ABCE1 containing a FLAG tag (DYKDDDDK) at the N terminus or left untransfected. After 24 h, cells were lysed in RIPA buffer and clarified lysates were separated by SDS-PAGE and transferred to PVDF membranes. Western blot analyses were performed with either the rabbit anti-ABCE1 serum (right panel) or a monoclonal mouse anti-FLAG antibody (left panel); secondary antibodies against either rabbit or mouse, respectively; and a mouse monoclonal antibody against β-actin directly conjugated to HRP as an internal control. (B) Reduction of ABCE1 mRNA levels following ABCE1_5 or ABCE1_6 siRNA transfection. ABCE1 mRNA copy numbers were quantified by real-time RT-PCR 48 h after siRNA transfection. ABCE1 mRNA copy numbers from three independent replicates were quantified. Error bars represent the standard deviation. (C) Continuous ABCE1 knockdown following ABCE1_5 or ABCE1_6 siRNA transfection. Reduction in cellular ABCE1 protein levels 48 h (left panel), 96 h (center panel), and 120 h (right panel) after siRNA transfection. ABCE1 bands from three independent replicates were quantified and normalized relative to an internal GAPDH control for each blot and are shown as percent reduction of ABCE1 expression relative to the NSC for each time point. Error bars represent the standard deviation. (D) Specificity of ABCE1_5 or ABCE1_6 siRNAs. A549-hSLAM cells were transfected with the FLAG-tagged ABCE1 expression plasmid or a derivative carrying 7 to 10 noncoding mutations in the binding sites of ABCE1_5 and ABCE1_6 siRNAs. After 48 h, the cells were transfected with the respective siRNAs, and ABCE1 protein levels were visualized by Western blotting using a FLAG-specific antibody 48 h later. (E) Validation of siRNA target specificity. Cells were transfected with the respective ABCE1 siRNAs or their corresponding C911 control variants. After 48 h, cells were infected with MeV at an MOI of 0.01. ABCE1 levels in uninfected cells are shown in the left panel, and MeV N levels in MeV-infected cells are shown in the right panel. Protein levels from three independent replicates were quantified, and the NSC value was set to 100%. Error bars represent the standard deviation. *, *P* < 0.05; **, *P* < 0.01; ***, *P* < 0.001.

We selected ABCE1 for further study since it was found to be a common proviral factor for MeV, MuV, and HRSV in our screens as well as for HeV (43). To reliably detect ABCE1, we generated a rabbit antipeptide hyperimmune serum targeting the 19 C-terminal amino acid residues of human ABCE1 and confirmed its specificity using a FLAG-tagged ABCE1 expression plasmid (Fig. 3A). Since generation of stable ABCE1 knockout cells failed repeatedly, and prolonged ABCE1 knockdown resulted in reduced cell proliferation, transient siRNA knockdown was used for all experiments. We then validated the extent of ABCE1 protein knockdown using the most effective siRNAs, ABCE1_5 and ABCE1_6. For both siRNAs, ABCE1 mRNA copy number was dramatically reduced, and protein expression levels were decreased by more than 60% (Fig. 3B and C), illustrating specificity and durability of the knockdown. Cells transfected with a plasmid encoding an siRNA-resistant ABCE1 protein or C911 control siRNAs retained wild-type ABCE1 and MeV N protein levels (Fig. 3D and E), demonstrating that the observed proviral effect is ABCE1 specific.

**FIG 4:**
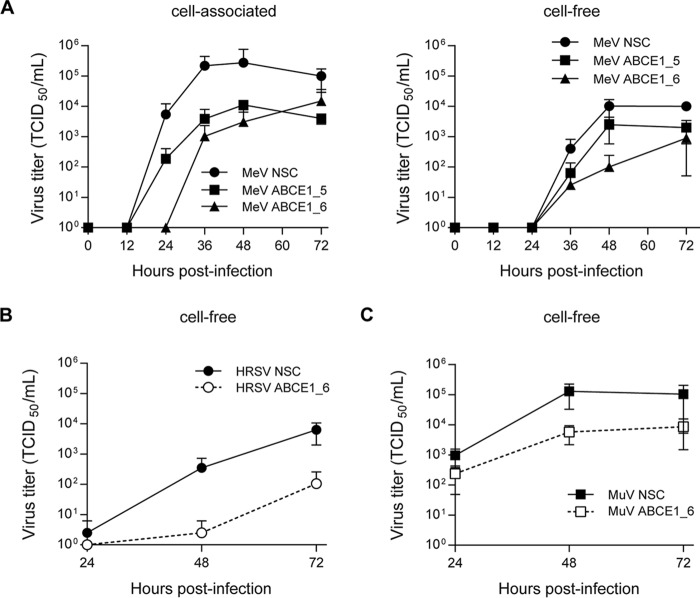
Validation of ABCE1 knockdown effect on MeV, MuV, and RSV replication. Reduction in virus replication as a result of ABCE1 knockdown. (A) A549-hSLAM cells were transfected with control NSC, ABCE1_5, or ABCE1_6 siRNAs for 48 h. Cells were then infected with MeV at an MOI of 0.01. Cell lysates (left panel) and culture supernatants (right panel) were harvested at the indicated time points and titrated on A549-hSLAM cells. (B and C) A549-hSLAM cells were transfected with control NSC or ABCE1_6 siRNAs for 48 h. Cells were then infected with either RSV or MuV at an MOI of 0.01. Supernatant titers are shown for RSV (B) and MuV (C). Each data point represents at least three replicates, and error bars indicate the standard deviation.

To evaluate the impact of ABCE1 on the viral life cycle, A549-hSLAM cells were transfected with either nonsilencing control (NSC), ABCE1_5, or ABCE1_6 siRNAs. After 48 h of incubation, the cells were infected with MeV at an MOI of 0.01 for a multistep growth curve, mimicking the screen conditions, and cell-associated and released virus was quantified every 12 h for 72 h. In cells transfected with ABCE1_6, production of cell-associated progeny virus was delayed by 12 h, and siRNA ABCE1_5 reduced virus production more than 10-fold. At later time points, titers in cells with reduced ABCE1 levels remained around 100-fold lower than in NSC siRNA-treated cells (Fig. 4A, left panel). While released virus was first detected after 36 h regardless of ABCE1 levels, a similar reduction in virus titers was observed, with siRNA ABCE1_6 having the strongest effect (Fig. 4A, right panel). HRSV displayed a similar dependency on ABCE1 (Fig. 4B), whereas for MuV, only around a 10-fold reduction in viral titers was observed (Fig. 4C). Taken together, these findings demonstrate that the reduction of cellular ABCE1 levels correspondingly reduces MeV, MuV, and HRSV replication, confirming ABCE1 as a biologically relevant host factor for *Paramyxoviridae* and *Pneumoviridae*.

### ABCE1 acts at the level of viral protein accumulation.

**FIG 5:**
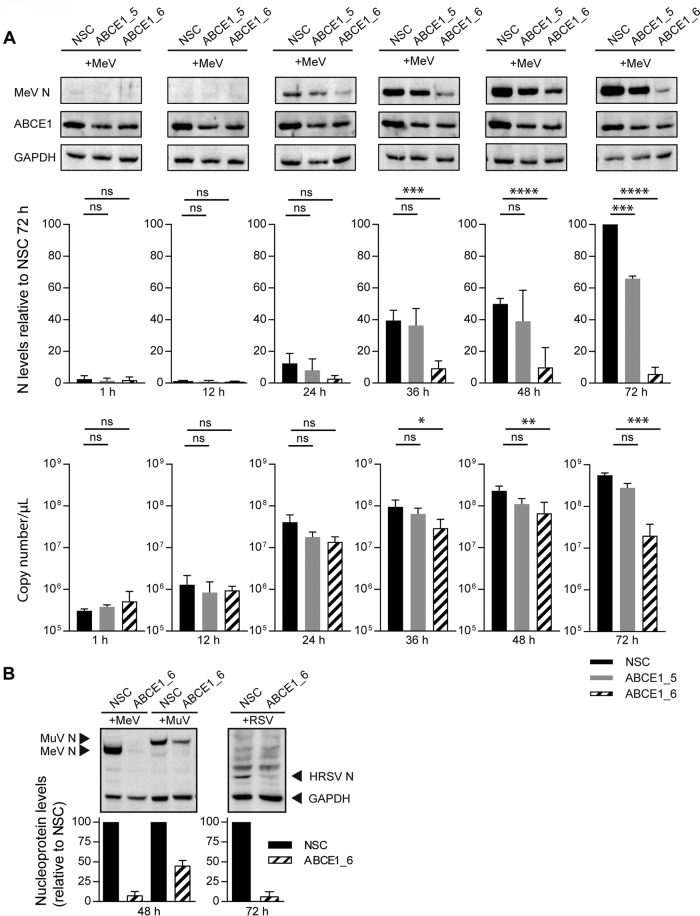
Assessment of ABCE1 knockdown on viral protein and mRNA levels. (A) Kinetics of N protein (top and middle panels) and mRNA (bottom panel) levels over the course of MeV infection at an MOI of 0.01. A549-hSLAM cells were transfected with control NSC, ABCE1_5, or ABCE1_6 siRNAs for 48 h. Cells were then infected, and samples were harvested at the indicated time points. N protein bands from three independent replicates were quantified and normalized relative to an internal GAPDH control for each blot and are shown as percent reduction of N protein expression relative to the NSC for each virus. Copy numbers of N gene mRNA were quantified from the same samples by real-time RT-PCR using a synthetized RNA standard. (B) Reduction of viral N protein expression in siRNA-transfected cells infected with either MeV or MuV (left panel) or RSV (right panel) at either 48 or 72 h postinfection, respectively. MeV, MuV, and RSV N protein bands from three independent replicates were each quantified and normalized relative to an internal GAPDH control for each blot and are shown as the percent reduction of viral N protein expression relative to the NSC for each virus. Error bars represent the standard deviation. ns, *P* ≥ 0.05; *, *P* < 0.05; **, *P* < 0.01; ***, *P* < 0.001; ****, *P* < 0.0001.

We then sought to characterize the mechanism underlying the proviral effect of ABCE1. No redistribution of ABCE1 in MeV-infected cells (Fig. S2A) and no colocalization of constituents of the viral ribonucleoprotein (RNP) complex or envelope with ABCE1 was observed (Fig. S2B). This suggests that ABCE1 does not interact with viral proteins during viral mRNA transcription, genome replication, or virion packaging, all of which occur in close association with the viral RNP. To investigate how ABCE1 affects viral RNA and protein synthesis, we followed MeV N protein and mRNA kinetics in NSC, ABCE1_5, or ABCE1_6 siRNA-transfected cells, which were infected after 48 h with an MOI of 0.01. Consistent with the growth kinetics (Fig. 4A), N protein was first detected at 24 h after infection, and expression levels consistently increased in control cells (Fig. 5A). Only minimal N protein levels were detected in siRNA ABCE1_6-treated cells throughout the experiment, whereas siRNA ABCE1_5 resulted in an intermediate phenotype (Fig. 5A). N protein mRNA levels remained stable for the first 12 h after infection and then increased around 100-fold within the next 12 h irrespective of ABCE1 levels, reflecting the first round of replication (Fig. 5A). There was a gradual increase in mRNA levels as the infection spread to new cells, which was less pronounced in ABCE1 siRNA-treated cells, resulting in statistically significant differences starting at 48 h after infection. A similar effect on viral proteins was observed with HRSV and MuV. Compared to the levels in NSC-transfected cells, the HRSV N protein was also barely detectable in the absence of ABCE1, and MuV N protein expression was reduced by more than 50% (Fig. 5B), which correlated with the impact of ABCE1 knockdown on the replication efficiency of these viruses (Fig. 4B and C), suggesting that ABCE1 plays an important role in the accumulation of viral proteins during infection.

10.1128/mBio.00826-19.2 FIG S2 Lack of direct interaction between ABCE1 and MeV proteins. (A) Distribution of ABCE1 in uninfected and MeV-infected cells. A549-hSLAM cells were infected with EGFP-expressing MeV at an MOI of 1 or left uninfected and then were fixed and permeabilized 17 h after infection and stained with rabbit anti-ABCE1 serum and Alexa Fluor 568-conjugated secondary antibody. Cells were visualized by fluorescence microscopy (magnification, ×400). Bar, 25 μm. (B) Costaining of ABCE1 and measles virus N, M, and H proteins. MeV-infected cells were fixed and permeabilized 24 h after infection and then stained with rabbit anti-ABCE1 serum and mouse monoclonal antibodies against either MeV N, M, or H, followed by Alexa Fluor 568-conjugated anti-rabbit and Alexa Fluor 488-conjugated anti-mouse secondary antibodies. Stained cells were imaged by confocal microscopy (magnification, ×1,000). Bar, 10 μm. Download FIG S2, PDF file, 7.8 MB. Copyright © 2019 Anderson et al. 2019 Anderson et al. This content is distributed under the terms of the Creative Commons Attribution 4.0 International license.

### Viral mRNA transcription is ABCE1 independent.

**FIG 6:**
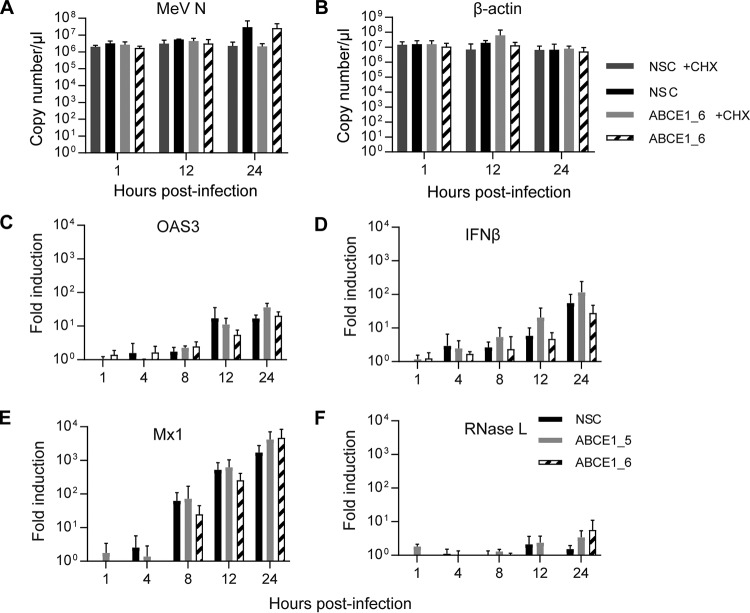
Role of ABCE1 in mRNA transcription and innate immune activation. (A and B) Analysis of *de novo* mRNA synthesis. A549-hSLAM cells were transfected with control NSC or ABCE1_6 siRNAs for 48 h, infected with MeV at an MOI of 1, and treated with 100 μg/ml CHX or left untreated starting 30 min before infection. Copy numbers of MeV N (A) and β-actin mRNA levels (B) were quantified at different times after infection by real-time RT-PCR using a synthetized RNA standard. (C to F) Evaluation of innate immune response activation. Induction of interferon-related gene expression was assessed by quantifying relative mRNA levels of OAS3 (C), IFN-β (D), Mx1 (E), and RNase L (F). Relative changes in mRNA levels from three independent experiments were quantified by real-time RT-PCR using the ΔΔ*C_T_* method with GAPDH as standard and are shown relative to the NSC signal at 1 hpi for each cytokine. Error bars indicate the standard deviation, and none of the differences seen reached statistical significance (*P* > 0.05).

To determine if the observed reduction in viral protein production is due to a direct role of ABCE1 in translation or a consequence of a direct or indirect involvement in viral mRNA transcription, we first compared viral and cellular mRNA levels in the presence and absence of cycloheximide (CHX). Cells transfected with control or ABCE1_6 siRNA were infected 48 h later with MeV at an MOI of 1 and then treated with CHX, which blocks *de novo* protein synthesis. We observed a gradual increase but no differences between copy numbers of MeV N mRNA in the presence or absence of cycloheximide for the first 12 h after infection (Fig. 6A), which is expected for the initial phase of viral mRNA transcription in infected cells driven by the viral polymerase complex provided in *trans* by incoming virions. Consistent with the kinetics at an MOI of 0.01 (Fig. 5A), mRNA copy numbers increased more than 10-fold in the absence of CHX at the 24-h time point, irrespective of cellular ABCE1 levels (Fig. 6A), whereas CHX treatment had no effect on β-actin mRNA levels (Fig. 6B). Taken together, this demonstrates that ABCE1 does not directly influence viral mRNA synthesis.

Since ABCE1 was first identified as an RNase L inhibitor (28), we initially attempted to investigate the effect of siRNA-mediated RNase L knockdown on the proviral effect of ABCE1. However, the RNase L protein levels in A549-hSLAM cells were not reliably detectable, leaving us unable to determine knockdown efficiency. We thus investigated if RNase L indirectly affects viral replication either by enhancing MeV replication in the presence of type I interferon (IFN) treatment or by inhibiting innate immune activation. Type I IFN treatment had little effect on MeV N protein levels in the presence or absence of ABCE1, indicating that virus replication is not affected (Fig. S3). Quantification of relative mRNA induction over the first 24 h after infection with an MOI of 1 revealed a gradual increase of 2′-5′-oligoadenylate synthase 3, the upstream activator of RNase L, IFN-β, and the IFN-stimulated gene Mx1 (Fig. 6C to E), which mirrored the increase in MeV N mRNA levels over the same period (Fig. 6A). While there was some variability, there was no correlation with ABCE1 levels in the cells (Fig. 6C to E). RNase L mRNA levels were also not affected and remained largely stable, with a slight increase at the 24-h time point (Fig. 6F). These results and the lack of an effect on viral RNA accumulation indicate that ABCE1 does not support *Paramyxoviridae* and *Pneumoviridae* replication by modulating global innate immune activation.

10.1128/mBio.00826-19.3 FIG S3 Type I interferon treatment does not affect MeV replication in the absence of ABCE1. (A) A549-hSLAM cells were transfected with either NSC, ABCE1_5, or ABCE1_6 siRNAs and were then pretreated with IFN-α (5 U/ml) 24 h later or left untreated. After 24 h, cells were infected with MeV at an MOI of 1. Cell lysates were harvested 24 h after infection. (B) The MeV N protein levels in panel A were quantified, and NSC was set to 100% for both untreated and IFN-treated cells. Data are representative of three independent replicates. Download FIG S3, PDF file, 0.8 MB. Copyright © 2019 Anderson et al. 2019 Anderson et al. This content is distributed under the terms of the Creative Commons Attribution 4.0 International license.

### ABCE1 is essential for efficient viral protein synthesis.

**FIG 7:**
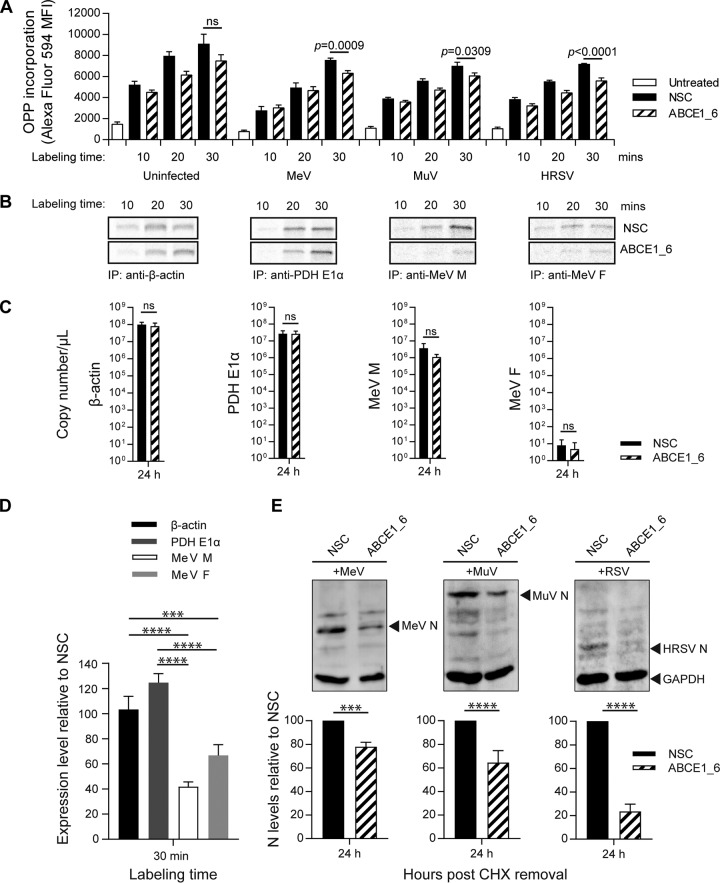
ABCE1 effect on *de novo* viral protein translation. (A) Quantification of total cellular protein synthesis by incorporation of *O*-propargyl-puromycin (OPP). At 48 h after siRNA transfection, cells were infected with MeV, MuV, or HRSV for a further 48 h, after which the cells were treated with OPP for the indicated times, and incorporated OPP was coupled to Alexa Fluor 594. The fluorescence signal was quantified by flow cytometry, and bars show the mean fluorescence intensity (MFI). Error bars represent the standard error of the mean. Statistical significance was determined by one-tailed *t* tests. (B to D) Analysis of protein translation by ^35^S pulse-labeling and quantification of the corresponding mRNA levels. A549-hSLAM cells were transfected with control NSC or ABCE1_6 siRNAs for 48 h. Cells were then infected with MeV at an MOI of 0.1 and labeled with [^35^S]Met-Cys 24 h later. (B) Immunoprecipitated cellular proteins β-actin and PDH E1α and MeV M and F proteins from infected cells were visualized by exposure to phosphor screens. Representative phosphor screen images are shown. (C) Copy numbers of the respective mRNAs from three independent experiments corresponding to the ^35^S pulse-labeling were quantified by real-time RT-PCR using a synthetized RNA standard for each gene. (D) Quantification of ^35^S pulse-label incorporation. The expression levels in samples shown in panel B from ABCE1_6 siRNA-treated cells relative to NSC-treated cells were calculated for each protein. The average from three independent experiments for the 30-min time point is shown. (E) Analysis of *de novo* protein synthesis of MeV, MuV, and RSV. A549-hSLAM cells were transfected with control NSC or ABCE1_6 siRNAs for 48 h; infected with MeV, MuV, or HRSV at an MOI of 1; and treated with 100 μg/ml CHX starting 30 min before infection. After 24 h, CHX was removed and samples were harvested after an additional 24 h of incubation. MeV, MuV, and RSV N protein bands were quantified and normalized relative to an internal GAPDH control for each blot and are shown as the percent reduction of viral N protein expression relative to the NSC for each virus. Each graph shows the average from three independent experiments, and error bars represent the standard deviation. Statistical significance in panels C to E is indicated as follows: ns, *P* ≥ 0.05; ***, *P* < 0.001; ****, *P* < 0.0001.

Given the effects on viral protein accumulation and the fact that ABCE1 is known to function in ribosome recycling (23, 24), we evaluated the relative importance of ABCE1 in cellular and viral protein translation, using catalyzed fluorescent labeling of proteins in cells treated with *O*-propargyl-puromycin (OPP). We found that the siRNA-mediated knockdown of ABCE1 resulted in a modest reduction of global cellular *de novo* protein synthesis in noninfected and infected cells. In the context of infection, this reduction reached statistical significance at longer labeling times (Fig. 7A), indicating a cumulative effect.

Knockdown of ABCE1 had no effect on protein synthesis of β-actin and pyruvate dehydrogenase (PDH) E1α, two cellular proteins with moderate and high turnover rates, respectively, whereas viral protein synthesis represented by the MeV M and fusion (F) proteins was reduced by 30 to 50% compared to the expression levels achieved in the presence of ABCE1 at the respective time points (Fig. 7B). Especially for the MeV F protein, which is present at smaller quantities due to the transcriptional gradient, the difference in the amount of newly synthetized protein increased over time (Fig. 7B, far right panel). No significant difference in cellular or viral mRNA levels was observed at the time point of the analysis (Fig. 7C), confirming that ABCE1 exerts its effect at the translational level. Comparison of cellular and viral protein expression levels in cells transfected with NSC or ABCE1_6 siRNA at the 30-min time point revealed around a 60% reduction for MeV M and a 40% reduction for MeV F, which was statistically significantly different to the cellular proteins (Fig. 7D). To assess the importance of ABCE1 for *Paramyxoviridae* and *Pneumoviridae* protein translation, we performed a CHX chase experiment. Cells were transfected with NSC and ABCE1_6 siRNA and then treated with 100 μg/ml CHX starting 30 min before infection with MeV, MuV, or HRSV at an MOI of 1. After 24 h, CHX was removed, and samples were collected 24 h later. Reduction of ABCE1 levels resulted in a significant inhibition of *de novo* synthesis of viral proteins (Fig. 7E), demonstrating a specific role of ABCE1 in translation of *Paramyxoviridae* and *Pneumoviridae* mRNAs.

### The ABCE1 proviral effect requires viral gene expression in the context of infection.

**FIG 8:**
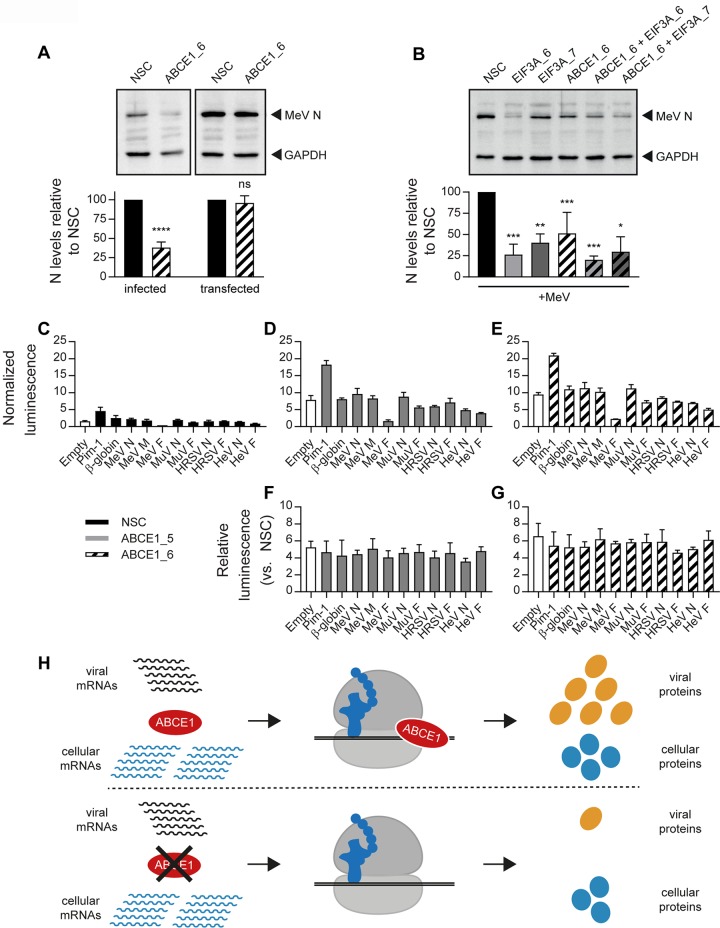
Dependence of ABCE1 proviral effect on viral gene expression during infection. (A) Plasmid-expressed MeV N is not sensitive to ABCE1 knockdown. A549-hSLAM cells were transfected with NSC or ABCE1_6 siRNAs. After 24 h, cells were transfected either with a control empty vector plasmid followed by infection with MeV at an MOI of 0.01 at 24 h posttransfection (“infected”) or with a plasmid expressing MeV N containing an N-terminal FLAG tag and left uninfected 24 posttransfection (“transfected”). The MeV N protein levels were quantified, and NSC was set to 100%. Data are representative of four replicates. Error bars represent standard deviations. Statistical significance is indicated as follows: ns, *P* ≥ 0.05; ****, *P* < 0.0001. (B) ABCE1 and eIF3A have similar effects on MeV replication. A549-hSLAM cells were transfected with the respective siRNAs. After 48 h, cells were infected with MeV at an MOI of 0.01. Cell lysates were harvested 48 h after infection. MeV N protein levels were quantified, and NSC was set to 100%. Data are representative of three independent replicates. ns, *P* ≥ 0.05; *, *P* < 0.05; **, *P* < 0.01; ***, *P* < 0.001. (C to G) Effect of viral 5′ untranslated regions (UTRs) on reporter gene translation. Hep-G2 cells were transfected with either NSC or the ABCE1_5 and ABCE1_6 siRNAs in 96-well plates along with dual-luciferase reporter plasmids. Translation of firefly luciferase (ffLuc) is mediated by upstream cellular or viral UTRs, and the translation of the downstream *Renilla* luciferase (RenLuc) is driven by the hepatitis C virus (HCV) internal ribosomal entry site (IRES). Luciferase values were read 48 h after transfection. ffLuc signals for each well were normalized to the RenLuc signal. Normalized ffLuc values are shown for cells transfected with NSC (C), ABCE1_5 (D), and ABCE1_6 (E), and the signals from ABCE1_5- and ABCE1_6-transfected cells are shown relative to NSC (F and G, respectively). Transfections in each replicate were performed in triplicate, and all experiments were performed three times. (H) Model for the differential effects of ABCE1 knockdown on viral and cellular protein translation. In cells with normal ABCE1 levels, viral proteins are preferentially translated over cellular proteins, resulting in proportionately higher levels of viral protein expression (top). In cells with reduced ABCE1 levels, viral protein translation is drastically reduced, while translation of cellular proteins is only moderately affected (bottom).

To further elucidate the virus-host interactions underlying the proviral activity of ABCE1, we investigated if the effect could be reproduced if MeV N protein was expressed from a cellular expression plasmid. In contrast to the reduction of MeV N protein levels observed in the absence of ABCE1, there was no effect on MeV N protein levels in cells transfected with a eukaryotic MeV N expression plasmid (Fig. 8A). Comparison of the proviral activity of ABCE1 with eIF3A, another hit in our validation screen and constituent of the cellular translation machinery, revealed a similar reduction in MeV N protein levels and no additive effect when the two genes were targeted simultaneously (Fig. 8B), highlighting the exquisite dependence of *Paramyxoviridae* and *Pneumoviridae* on specific translation factors.

Since the 5′ untranslated regions (UTRs) of paramyxoviral and pneumoviral mRNAs can vary in length depending on the gene and virus (3), sensitivity to ABCE1 knockdown was assessed. Toward this, the 5′ UTRs of the MeV N, M, and F genes, as well as those from the MuV, HRSV, and HeV N and F genes, were introduced upstream of a firefly luciferase (ffLuc) reporter gene followed by a hepatitis C virus (HCV) internal ribosomal entry site (IRES) that mediates translation of a *Renilla* luciferase (RenLuc) reporter gene for normalization (48). While ABCE1 knockdown resulted in an overall increase in ffLuc expression from all the tested plasmids independently of the 5′ UTR inserted (Fig. 8C to E), the relative expression levels remained stable, demonstrating that viral 5′ UTRs were not sufficient to confer sensitivity to ABCE1 knockdown (Fig. 8F and G). Taken together, our results illustrate that *Paramyxoviridae* and *Pneumoviridae* mRNAs are disproportionally sensitive to loss of ABCE1 function (Fig. 8H). Perturbations in ABCE1 levels are tolerated by the cell but have a dramatic effect on the viral life cycle.

## DISCUSSION

Even though nonsegmented negative-strand RNA viruses that replicate in the cytoplasm exploit the host cell machinery for many aspects of their life cycles, the cellular proteins and networks involved in these interactions remain largely unknown (49). Here, we performed comparative whole-genome siRNA screens with wild-type MeV, MuV, and HRSV strains in a human lung alveolar epithelial carcinoma cell line as a representative target cell type. The meta-analysis of two independent comparative analyses of the three siRNA screens highlighted 24 candidates that were validated experimentally. Six of these genes, ABCE1, COPB2, RBM22, FOXP4, HSD11B2, and PRR15, were also hits in a recently published HeV screen (43). Detailed characterization of ABCE1, a multifunctional protein involved in inhibition of RNase L-mediated signaling, HIV particle assembly, and ribosome recycling (50), revealed that it plays a critical role in viral protein synthesis.

Previous screens using HeV and VSV in HeLa cells identified COPI and other proteins involved in retrograde vesicular transport from the Golgi complex to the endoplasmic reticulum as a common proviral pathway for all negative-stranded RNA viruses (10, 43). While the function of this pathway in the biology of negative-stranded RNA viruses remains to be characterized, it likely supports replication analogous to its role in the life cycle of positive-stranded RNA viruses (51, 52). In addition to confirming the importance of this pathway, our screens also highlighted a prominent role for oxidative phosphorylation. Mitochondria are at the crossroads of different cellular antiviral defense mechanisms, and the importance of the pathway has been identified for different virus families (53). For influenza virus, NOX4-mediated increase of reactive oxygen species (ROS) is required for efficient replication by supporting the nuclear export of RNPs and particle formation and budding (54). In HRSV, phosphorylated p38 mitogen-activated protein kinase was found to be sequestered in viral inclusion bodies (55), and it is thus conceivable that such sequestration also occurs with other paramyxoviruses.

ABCE1 is a member of the superfamily of ABC transporters that contain two nucleotide-binding domains and two N-terminal iron-sulfur clusters. It is ubiquitously expressed at high levels in the trachea, testis, and prostate (56). Unlike most ABC domain proteins, members of the ABCE subfamily do not contain the membrane-spanning domains and may thus not act as transporters (21). ABCE1 was initially identified as a negative regulator of the interferon-induced 2-5A antiviral pathway, where it functions by blocking RNase L (28). RNase L plays an important role in the antiviral and antiproliferative activities of IFN and contributes to innate immunity and cell metabolism (57, 58). ABCE1 in turn supports the replication of several RNA viruses by inhibiting the IFN-induced activation of the 2-5A/RNase L pathway. During West Nile virus infection, inhibition of ABCE1 results in cleavage of viral genomic RNA by RNase L, indicating that RNase L plays a role in the cellular antiviral response to flaviviruses (59). Furthermore, the potential of RNase L activator drugs to block paramyxovirus infection was demonstrated by administration of a 2-5A homolog to reduce HRSV replication in African green monkeys (60). Small-molecule activators of RNase L have also been shown to have antiviral activity against Sendai virus and human parainfluenza virus 3 (61), highlighting the importance of this pathway. Additionally, OASL, which was identified in our screens as a potential antiviral factor by the KS analysis (see Table S5 in the supplemental material), is a known antiviral effector for HRSV and is countered by the NS1 protein (62). However, our cytokine mRNA induction kinetics revealed that cellular ABCE1 levels do not affect global innate immune signaling pathways, and levels of MeV mRNAs were not altered in ABCE1-depleted cells. Nonetheless, we cannot formally rule out that the RNase-L-inhibitory activity of ABCE1 plays a minor role as a proviral factor for *Paramyxoviridae* and *Pneumoviridae*.

ABCE1 is involved in the regulation of translation (22) and as a ribosome-recycling factor critical for translation termination (63, 64) and ribosome homeostasis (23, 24). In contrast to many viruses which induce a host cell shutoff by interfering with cellular mRNA or protein synthesis, replication of *Paramyxoviridae* and *Pneumoviridae* seems to require ongoing cellular protein synthesis: Parainfluenza virus 5 P and V proteins actively prevent host gene shutoff by limiting PKR induction (65), and a similar effect was also reported for the MeV C protein (66). HRSV infection results in PKR induction, but the protein is sequestered by binding to the viral N protein, thereby attenuating the phosphorylation of eIF2α and maintaining cellular protein translation (67). Our data, however, suggest that MeV, MuV, and HRSV infections lead to a modest decrease in global proteins synthesis (Fig. 7A). Most importantly, and consistent with previous reports about the role of ABCE1, we observed a modest reduction in overall protein synthesis in ABCE1 knockdown cells. In contrast, viral protein synthesis was dramatically affected under the same conditions, demonstrating that this process is critically dependent on ABCE1. It is interesting that ABCE1 is known to form a stable complex with the small ribosomal subunit during the recycling of terminated or stalled ribosomes, and in our work 30 of the small ribosomal subunit proteins were among the top 100 proviral gene products predicted by the KS analysis (Table S2). In contrast, only five proteins of the large subunit scored in the same group, even though an important role of the large ribosomal subunit protein rpL40 for negative-strand RNA virus translation, including MeV, has recently been demonstrated (68). This disproportionate dependence on small ribosomal subunit proteins is the exact opposite of what has been observed in screens for human flaviviral host factors, where large subunit proteins predominate at the top of the list (46). Additionally, both the KS analysis and the robust Z score analyses identified eIF3A as a top proviral factor and suggested that other eIF3 subunits were likely involved, implying that the eIF3 complex may be exquisitely required for at least some paramyxovirus mRNA translation. ABCE1 has been shown to interact with eIF3 in Saccharomycescerevisiae (69), and structural studies indicated that ABCE1 bound to the small ribosomal subunit prevents premature binding of eIF3 (63), thereby regulating the use of recycled ribosomes in further initiation events. Another hit common to all three viruses was RPLP1, which has recently been implicated in ABCE1-dependent ribosome recycling (70). These data strongly suggest that the paramyxovirus requirement for ABCE1 is due to its role in ribosome recycling.

Why are paramyxovirus mRNAs so sensitive to ABCE1? There is much we do not know about paramyxovirus translation, but certain facts point to special requirements for mRNAs encoded by these viruses. It must be emphasized that not all paramyxovirus and pneumovirus mRNAs may require ABCE1 to the same extent, since these mRNAs vary in their structure and likely in their dependence on host factors. MeV N mRNA translation was shown to be enhanced by La autoantigen (SSB) overexpression, and this was proposed to be mediated by an interaction between SSB and the 5′ UTR of MeV N mRNA (71). However, in this report the authors did not determine the effect of knocking down SSB on MeV propagation, and in our screens SSB did not score as either proviral or antiviral. One of the peculiar features of paramyxovirus mRNAs is that they can be polycistronic (72–76), which could lead to altered termination of ribosomes in the short segments between cistrons and thus a more stringent requirement for ABCE1. These polycistronic transcripts, even though they are a small fraction of all viral mRNAs, could act as reservoirs for recycling factors by trapping them in inactive complexes and thus increasing the requirement for ABCE1. Additionally, most paramyxovirus mRNAs, either monocistronic or polycistronic, have short 5′ and 3′ UTRs that again may interfere with efficient termination or use of recycled ribosomes for initiation. In a competitive translational landscape, where mRNAs are competing for a limited pool of ribosomes (77), viral mRNAs may be exquisitely dependent on the action of factors such as ABCE1.

## MATERIALS AND METHODS

### Cell lines and viruses.

A549 cells (ATCC CCL-185), Vero cells (ATCC CCL-81), and HeLa cells (ATCC CCL-2) constitutively expressing the human SLAM molecule were generated by selecting zeocin-resistant clones after transfection with pCG-IRESzeo-hSLAM, which was cloned as described for canine SLAM (78). All human SLAM (hSLAM)-expressing cell lines were maintained in Dulbecco modified Eagle medium (DMEM; Invitrogen) supplemented with 10% fetal bovine serum (FBS; Invitrogen) and 100 μg/ml zeocin (Invitrogen) at 37°C and 5% CO_2_. The human Epstein-Barr virus-transformed B-lymphoblastic cell line (BLCL) (W. P. Duprex laboratory [79]) and HEp-2 (ATCC CCL-23) and Hep-G2 cells (kindly provided by F. Elgner, Paul-Ehrlich-Institute) were maintained in RPMI supplemented with 10% FBS.

rMV^KS^EGFP(3) is a recombinant virus based on the Khartoum-Sudan (KS) strain of MeV (33, 80). rHRSV^B05^EGFP(5) is a recombinant virus based on the sequence of HRSV present in a tracheal rinse from an HRSV-positive infant during the 2004–2005 HRSV season (35). rMuV^G09^EGFP(3) is a recombinant virus based on the sequence of MuV present in clinical material from a genotype G MuV infection from the 2009 New York mumps outbreak (34, 81). The recombinant paramyxoviruses all express EGFP from an additional transcription unit inserted between the P and M genes; this is position 3 in the recombinant genome for MeV and MuV and position 5 for HRSV. MeV was propagated in BLCL cells while MuV and HRSV were amplified in HEp-2 cells. Prior to storage, sucrose (25% [wt/vol]) was added to HRSV stocks.

### Primary genome-wide siRNA screens.

The screens were performed at the Duke University Functional Genomics Facility using the Qiagen genome siRNA library v1.0 consisting of four distinct siRNAs (A, B, C, and D) originally designed to target 22,909 known and putative human genes. As many instances of obsolete or outdated library gene annotations were found in the original annotation, the siRNA library annotations were updated based on the perfect match of their sequence to the GRCh37v75 release of the human transcriptome, resulting in the targeting of 21,705 genes by the siRNA library. The four siRNAs were grouped into two pools, with each pool containing two siRNAs (set AB and set CD). Each screen consisted of 148 384-well plates. This 2-by-2 pool design allowed each gene to be tested by two independent siRNA sets. Briefly, black, clear-bottom 384-well tissue culture plates (Corning) were prearrayed with 1 pmol of siRNA per well by using the Velocity Bravo liquid handling system (Agilent Technologies). Lipofectamine RNAiMAX (Invitrogen) was used in the amount of 0.05 μl per well in 10 μl of Opti-MEM (Invitrogen). After 15 min of complex formation, cells were dispensed at a density of 1,500 cells per well using a Matrix WellMate dispenser (reverse transfection of 15.4 nM final concentration of siRNAs in 65-μl total medium volume). To facilitate gene knockdown by siRNAs, plates were incubated at 37°C at 5% CO_2_ for 48 h. Virus infections at different MOIs and for various lengths of time postinfection were conducted to identify suitable conditions for the high-throughput screens. Transfected cells were infected with 20 μl of virus diluted in DMEM and supplemented with 10% FBS and were incubated at 37°C in 5% CO_2_. Cells infected with MeV were incubated for 33 h, and those infected with MuV and HRSV were incubated for 72 h. To identify factors involved in all stages of the paramyxovirus life cycle, the screen was optimized to allow for multiple rounds of infection.

### Validation screen.

For validation of the top-ranking genes identified by comparative analysis of the three primary screens, multiple siRNAs targeting the respective genes were used, and siRNAs that differed from those used in the primary screens were chosen whenever available. The assay was conducted in 384-well plates using the same reverse transfection procedure outlined for the primary screen. Each individual siRNA was represented in replicates of 4 in each plate. Image collection and analysis were performed using the same methodology as the original screens.

### Automated image analysis.

After 33 h for MeV infection or 72 h for MuV and HRSV infection, cells were fixed with 4% paraformaldehyde (Sigma) in phosphate-buffered saline (PBS; Invitrogen), permeabilized with 0.1% Triton X-100 in PBS, and stained with Hoechst 33342 (Sigma) in PBS for 30 min. Stained cells were imaged and analyzed with a Cellomics ArrayScan VTI automated microscope. Uninfected cells served as a reference population for background fluorescence. Four fields per well of 384-well plates were imaged at ×10 magnification and analyzed using the Compartmental Analysis Bioapplication. First, images were acquired for Hoechst nuclear staining in channel 1 and EGFP signal in channel 2. Cells were identified and counted in channel 1, and cell numbers are indicated as VOC (valid object count). Infected cells were identified by EGFP signal in channel 2 and expressed as the percentage of total VOC.

### General statistical methods.

The distribution of percent infection for genomic population for the AB and CD pools did not follow a normal distribution, so we normalized raw percent infection measurements by converting to robust Z (82). Primary data from the Cellomics Oracle database were retrieved, and raw percent infection measurements were converted to robust Z scores within each plate using custom Python scripts. To identify candidate host factor and restriction genes, we evaluated the distribution of normalized well values for each gene relative to the overall distribution of all wells using the Kolmogorov-Smirnov (KS) test and used the false discovery rate (FDR) method to correct for multiple hypothesis testing. A similar approach to the gene-level analysis was employed to identify pathways enriched for either negative or positive selection. All pathways were collected from the “Curated Pathways” subset of the Molecular Signatures Database (MsigDB), and the KS test was used to evaluate the distribution of normalized values belonging to all genes of each pathway against the entire distribution of normalized values.

### Validation of ABCE1 as a host factor for *Paramyxoviridae* and *Pneumoviridae*.

A549-hSLAM cells were seeded into 24- or 12-well dishes and transfected with ABCE1-specific, eIF3A-specific, or control siRNAs. C911 siRNAs were used to control for off-target effects with the ABCE1 siRNAs. Target sequence mismatches were introduced into the ABCE1_5 and ABCE1_6 siRNAs using the C911 calculator version 1 (http://rnai.nih.gov/haystack/C911Calc.html) (83) to generate the ABCE1_5_C911 and ABCE1_6_C911 siRNAs, respectively (all from Sigma-Aldrich). Briefly, 100 or 200 μl/well of Opti-MEM (Invitrogen) was combined with 0.75 or 1.5 μl siRNA at a 20 μM working concentration and 1 or 2 μl of RNAiMAX transfection reagent (Invitrogen), vortexed, and incubated at room temperature for 15 min. The siRNA–Opti-MEM mixture was added to the plated A549-hSLAM cells, which were then incubated at 37°C. After 48 h, cells were infected with the respective viruses at an MOI of 0.01 or 1, and for CHX experiments, 100 μg/ml CHX was added starting 30 min before infection and maintained for the indicated duration. At different time points thereafter, the medium was harvested for virus titrations, and cells were lysed in RIPA buffer containing cOmplete protease inhibitor cocktail (Roche) for protein analysis or RNA isolation buffer. Culture supernatants were titrated by limiting dilution on A549-hSLAM cells and are expressed as the 50% tissue culture infectious dose (TCID_50_) per milliliter.

### MeV N expression plasmid transfection.

The plasmid pCG-MeV FLAG-N encodes the MeV N protein with a FLAG-tag (DYKDDDDK) at the N terminus. For transfection in siRNA-transfected A549-hSLAM cells in 12-well dishes, 2 μg of plasmid was mixed with 3 μl of Lipofectamine 2000 (Invitrogen) in 100 μl/well Opti-MEM (Invitrogen). Transfection mixtures were vortexed and incubated at room temperature for 20 min. After incubation, 100 μl of transfection mixture was added to the appropriate well and incubated at 37°C for 5 h, after which the medium was aspirated and replaced with fresh DMEM containing 5% FBS and 1% l-glutamine.

### Viral and cellular mRNA quantification.

Copy numbers of viral, β-actin, and PDH E1α mRNAs and relative expression levels of OAS3, IFN-β, Mx1, and RNase L in samples isolated at different times after infection of NSC or ABCE1 knockdown cells were quantified by real-time RT-PCR using the QuantiFast SYBR Green PCR kit (Qiagen) after reverse transcription with SuperScript III reverse transcriptase (Invitrogen). Each experiment was carried out at least three times.

### Antibodies.

For the detection of viral proteins by Western blot analysis or radiolabeling, mouse monoclonal antibodies against the MuV N protein (Abcam; ab9878), or the HRSV N protein (Abcam; ab94806) or rabbit antipeptide antisera against the MeV N, M, and F proteins, respectively (84), were used. Mouse monoclonal antibodies against actin (Abcam; ab8226), PDH E1α (Abcam; ab92696), and GAPDH (Cell Biolabs) and a rabbit antipeptide serum against ABCE1 (85) were used to visualize cellular proteins. The latter antiserum was kindly provided by Jaisri Lingappa (University of Washington, USA) for initial studies and subsequently produced by immunizing rabbits with the peptide N-NSIKDVEQKKSGNYFFLDD-C, corresponding to 19 amino acids at the C terminus of human ABCE1, coupled to keyhole limpet hemocyanin (GenScript). For immunofluorescence staining, monoclonal antibodies against MeV N (MAB8906), MeV M (MAB8910), or MeV H (MAB8905) proteins (all from Merck Millipore) were used. Horseradish peroxidase-coupled goat anti-mouse (Jackson ImmunoResearch) or goat anti-rabbit (Abcam; ab97200) secondary antibody and goat anti-mouse or goat anti-rabbit secondary antibodies conjugated to Alexa Fluor 488 and Alexa Fluor 568 (Molecular Probes) were used as secondary antibodies.

### Immunofluorescence staining.

A549-hSLAM cells were seeded on coverslips in 24-well plates and infected with MeV at an MOI of 1. After 1 h of infection, the culture medium was removed and replaced with medium including 20 μM Z-fFG (fusion inhibitory peptide [FIP]; Bachem). At 24 h postinfection, cells were fixed with 4% paraformaldehyde in PBS for 15 min and subsequently permeabilized with 0.1% Triton X-100 in PBS for 15 min at room temperature. Cells were stained with the respective primary and secondary antibodies, and coverslips were mounted onto glass slides with ProLong Gold antifade mountant with DAPI (Life Technologies). Images were obtained using a Zeiss LSM 510 confocal laser scanning microscope with a αPlanapochromat 100× oil objective. Images were collected at 1,008- by 1,008-pixel resolution and processed using ImageJ and Photoshop.

### Western blot analysis.

For each sample, the same protein concentration was loaded to correct for ABCE1 knockdown-mediated reduction in proliferation. Membranes were stained with the respective antibodies, proteins were visualized using a MicroChemi 4.2 chemiluminescent imaging system (DNR Bio Imaging Systems Ltd.), and the resulting bands were quantified from underexposed TIF images using the ImageJ analysis software package (NIH).

### Analysis of total *de novo* protein synthesis.

Cells were labeled with *O*-propargyl-puromycin (OPP; ThermoFisher) at different times after infection according to the manufacturer’s instructions. Briefly, after the respective labeling time, cells were fixed, OPP-labeled proteins were coupled to Alexa Fluor 594 picolyl azide using the Click-iT Plus OPP protein synthesis assay kit (ThermoFisher), and the Alexa Fluor 594 signal was assessed using a BD Accuri flow cytometer.

### Metabolic labeling assay.

Infected cells were labeled with 0.1 mCi (3.7 MBq) per well of [^35^S]Cys-Met mix (Hartmann Analytic). After the respective labeling time, the cells were lysed, divided in two samples, and mixed with protein A-agarose beads (Santa Cruz) and the respective antibody. After overnight incubation at 4°C, the proteins were eluted and separated on Mini-Protean 10% TGX gels (Bio-Rad). Dried gels were exposed to Kodak cassette-K BaFBr:Eu phosphor screens overnight and imaged using the Bio-Rad Personal Molecular Imager system. Bands were quantified using the Bio-Rad Quantity One 1-D Analysis software package.

### Interferon treatment.

Alpha interferon (IFN-α) stock solution (1.1 × 10^8^ U/ml; PBL Assay Science) was diluted to a final concentration of 5 U/ml in culture medium. siRNA-transfected cells were treated with IFN-α for 24 h before infection with MeV.

### Dual-luciferase UTR reporter assays.

Viral 5′ untranslated regions (UTRs) were cloned into the dual-luciferase reporter plasmid pFR_HCV_xb(leer) (48), kindly provided by A. Grünweller (Marburg, Germany). Transcription of the entire cassette is driven by the herpes simplex virus 1 (HSV-1) thymidine kinase (TK) promoter. The cap-mediated translation of the firefly luciferase (ffLuc) reporter is modulated by the viral UTR immediately upstream, while translation of the downstream *Renilla reniformis* luciferase (RenLuc) reporter used for normalization is mediated by the hepatitis C virus (HCV) internal ribosomal entry site (IRES).

The MeV N, MeV M, MuV F, HRSV N, HRSV F, and HeV N UTRs were generated by overlapping primer synthesis before being fused to the 5′ terminus of the firefly luciferase (ffLuc) reporter gene. The MeV F and MuV N UTRs were amplified from purified viral RNA and fused to the ffLuc 5′ terminus by three-segment overlapping PCR. The HeV F UTR was synthesized based on the GenBank reference sequence (accession number AF017149) and fused to the ffLuc 5′ terminus as for the MeV F and MuV N UTRs. The recombinant segments were cloned into the EcoRI and SphI sites of pFR_HCV_xb(leer) to generate the respective reporter plasmids.

For transfections, Hep-G2 cells were seeded at a density of 2 × 10^4^ cells/well in clear-bottom black 96-well plates in RPMI containing 10% FBS and 1% l-glutamine. Cells were reverse transfected in 96-well plates with the NSC, ABCE1_5, and ABCE1_6 siRNAs as described above for A549-hSLAM cells, as well as 10 μl/well of Opti-MEM containing 0.2 μg of the respective reporter plasmids mixed with 0.3 μl Lipofectamine 2000 for each well. Plates were then incubated at 37°C for 48 h. To quantify luciferase activity, the medium from each transfected well was aspirated and then replaced with 50 μl/well fresh RPMI plus 10% FBS. The ffLuc and RenLuc signals were then read using the Dual-Glo luciferase assay system (Promega) according to the manufacturer’s instructions. Fifty microliters/well each of the Dual-Glo reagent and the Dual-Glo Stop & Glo reagents was used to develop the ffLuc and RenLuc signal, respectively. Luciferase signals were read using a PheraStar luminometer. The ffLuc signal for each well was normalized to the internal RenLuc control signal, and the triplicate values from each reporter were averaged for each experiment. All experiments were performed at least three times.
