# Supplementary figures and images for: Comparative Loss-of-Function Screens Reveal ABCE1 as an Essential Cellular Host Factor for Efficient Translation of Paramyxoviridae and Pneumoviridae
Source: mBio. 2019 May 14;10(3):e00826-19. doi: 10.1128/mBio.00826-19 (PMC6520455; doi:10.1128/mBio.00826-19)

**Figure S1**

**A**

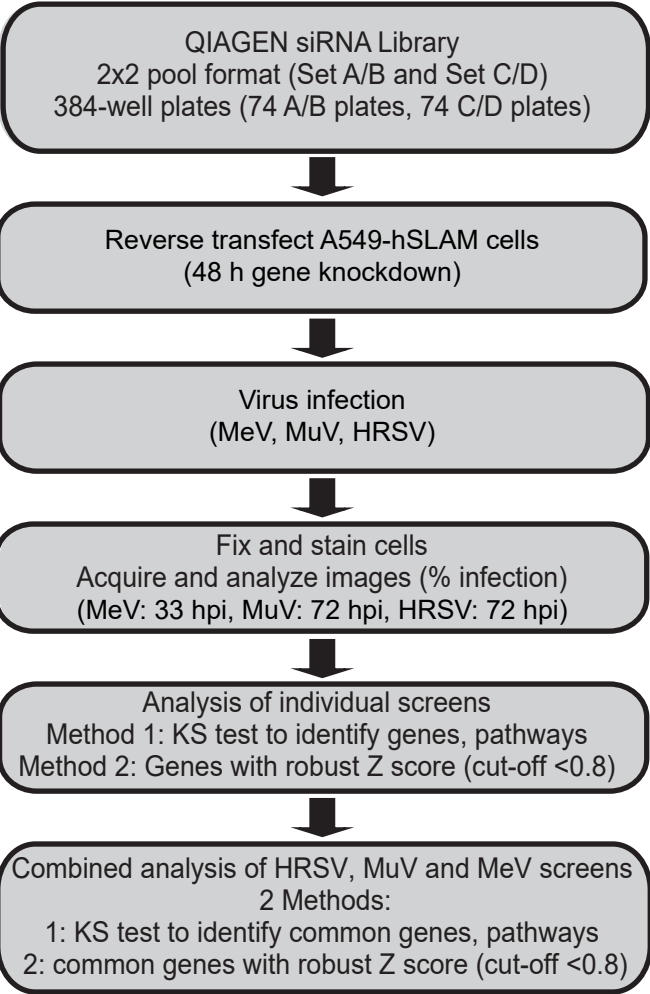

**B**

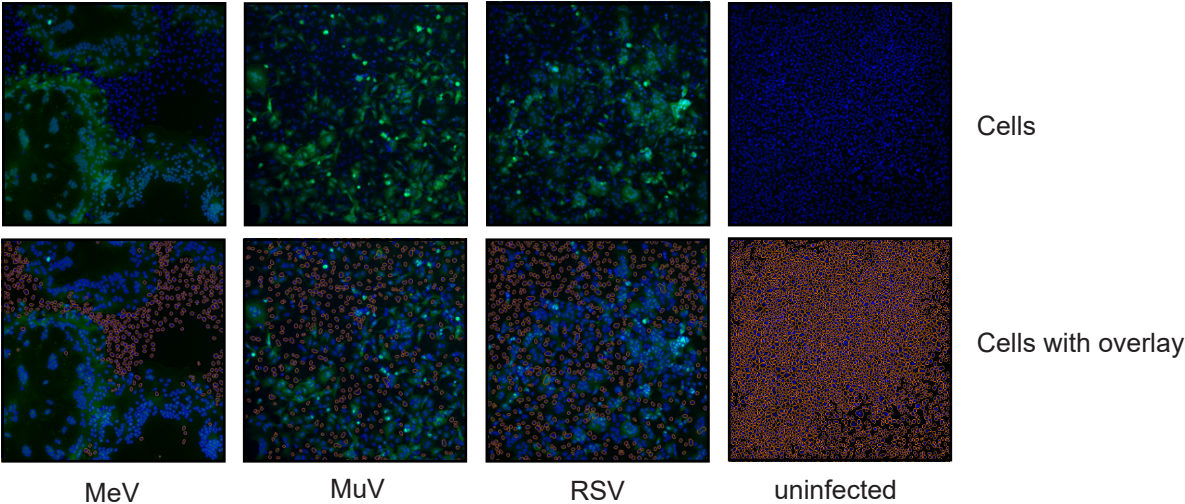

Supplement: FIG S1 [file mBio.00826-19-sf001.pdf]

**Figure S2**

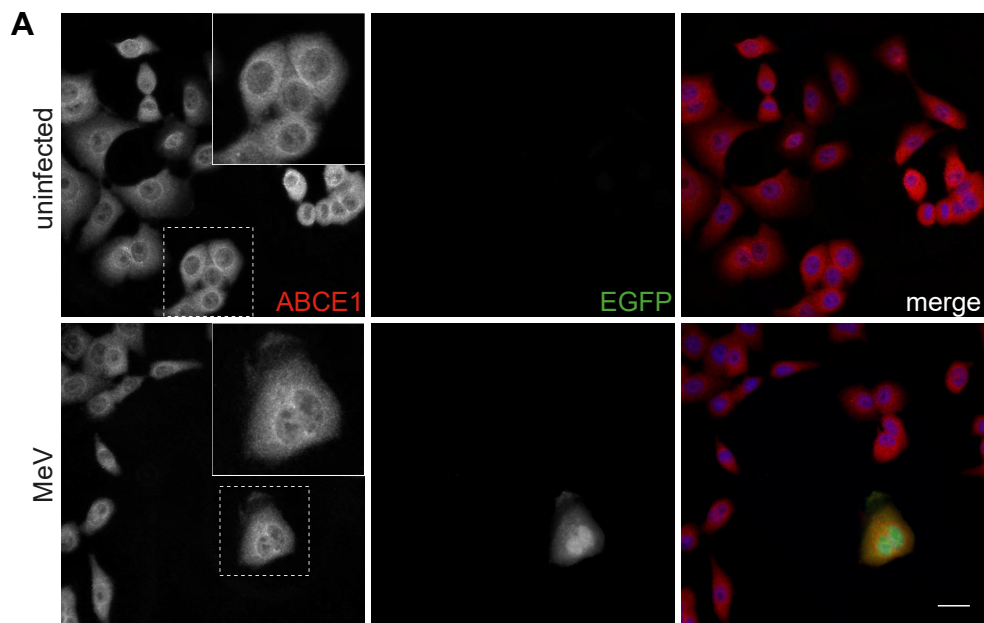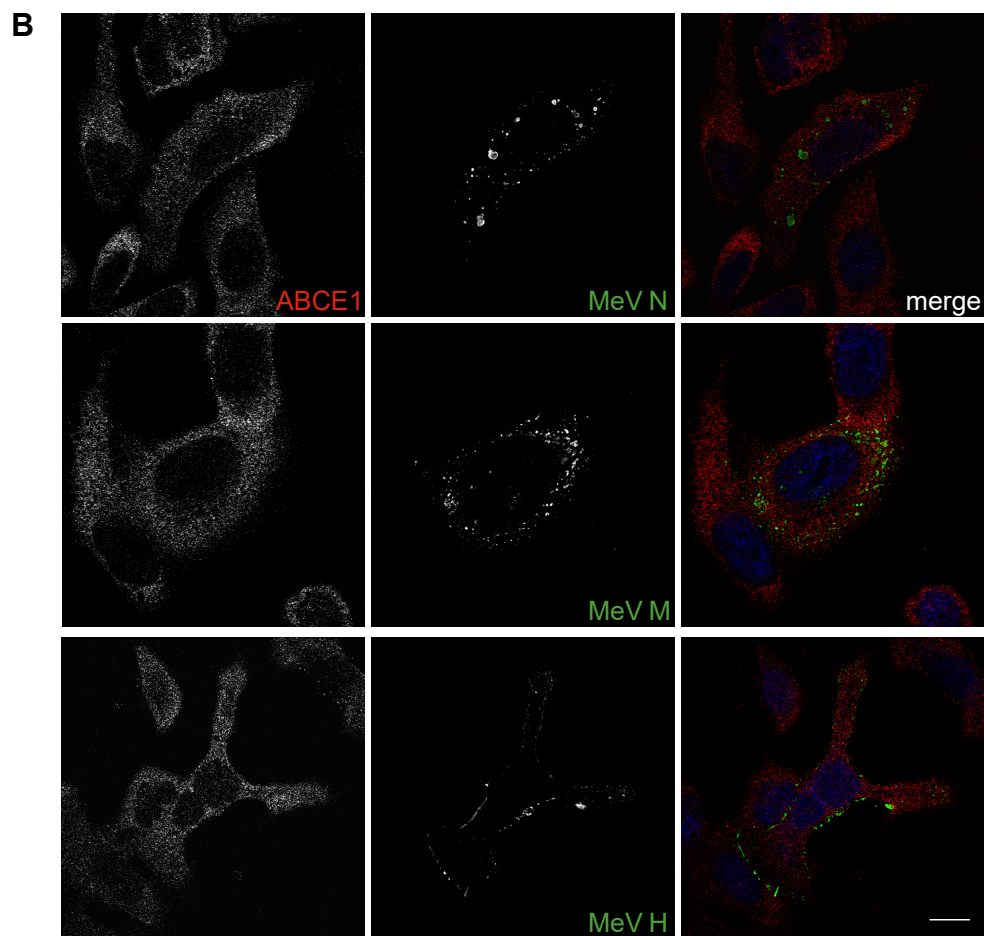

Supplement: FIG S2 [file mBio.00826-19-sf002.pdf]

Figure S3

A

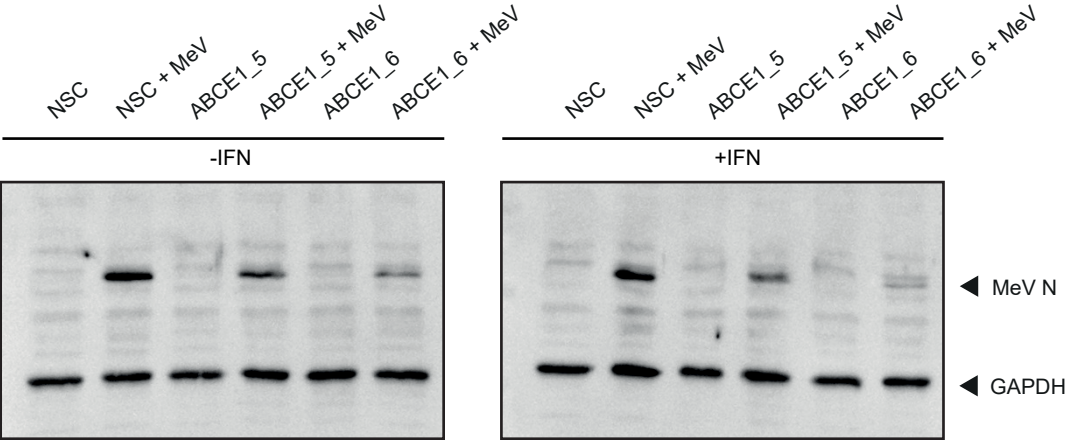

B

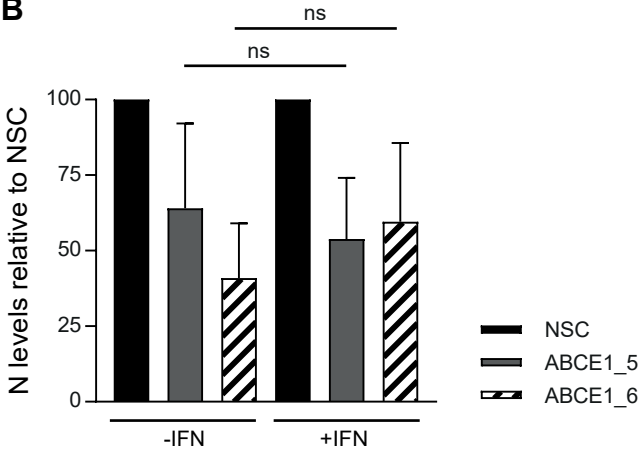

Supplement: FIG S3 [file mBio.00826-19-sf003.pdf]
