# Supplementary material for: Comparative Loss-of-Function Screens Reveal ABCE1 as an Essential Cellular Host Factor for Efficient Translation of Paramyxoviridae and Pneumoviridae
Source: mBio. 2019 May 14;10(3):e00826-19. doi: 10.1128/mBio.00826-19 (PMC6520455; doi:10.1128/mBio.00826-19)
Supplement: TABLE S5 [file mBio.00826-19-st005.pdf]

| Official HGNC Gene Symbol | Official HGNC Full Name | Entrez Gene ID | Present in KS analysis | Selected for validation |
|---|---|---|---|---|
| **ABCE1** | **ATP binding cassette subfamily E member 1** | **6059** | **yes** | **yes** |
| ATG9B | autophagy related 9B | 285973 | yes | |
| DOK7 | docking protein 7 | 285489 | yes | |
| **EIF3A** | **eukaryotic translation initiation factor 3 subunit A** | **8661** | **yes** | **yes** |
| HSPB7 | heat shock protein family B (small) member 7 | 27129 | yes | |
| PABPC5 | poly(A) binding protein cytoplasmic 5 | 140886 | yes | |
| **PRR15** | **proline rich 15** | **222171** | **yes** | **yes** |
| UXS1 | UDP-glucuronate decarboxylase 1 | 80146 | yes | |
| RPS9 | ribosomal protein S9 | 6203 | yes | |
| ARCN1 | archain 1 | 372 | yes | |
| C2 | complement component 2 | 717 | yes | |
| COPA | coatomer protein complex subunit alpha | 1314 | yes | |
| COPB1 | coatomer protein complex subunit beta 1 | 1315 | yes | |
| **COPB2** | **coatomer protein complex subunit beta 2** | **9276** | **yes** | **yes** |
| COPZ1 | coatomer protein complex subunit zeta 1 | 22818 | yes | |
| GOT1L1 | glutamic-oxaloacetic transaminase 1-like 1 | 137362 | yes | |
| **HSD11B2** | **hydroxysteroid (11-beta) dehydrogenase 2** | **3291** | **yes** | **yes** |
| IK | IK cytokine, down-regulator of HLA II | 3550 | yes | |
| KRT18P16 | keratin 18 pseudogene 16 | 391827 | yes | |
| NDUFB4P12 | NADH:ubiquinone oxidoreductase subunit B4 pseudogene 12 | 402175 | yes | |
| NME3 | NME/NM23 nucleoside diphosphate kinase 3 | 4832 | yes | |
| OR6C4 | olfactory receptor family 6 subfamily C member 4 | 341418 | yes | |
| **RBM22** | **RNA binding motif protein 22** | **55696** | **yes** | **yes** |
| RPLP1 | ribosomal protein lateral stalk subunit P1 | 6176 | yes | |
| AKTIP | AKT interacting protein | 64400 | | |
| COL13A1 | collagen type XIII alpha 1 | 1305 | | |
| COPG1 | coatomer protein complex subunit gamma 1 | 22820 | | |
| SUPT20HL1 | SPT20 homolog, SAGA complex component-like 1 | 100130302 | | |
| ACP7 | acid phosphatase 7, tartrate resistant (putative) | 390928 | | |
| **FOXP4** | **forkhead box P4** | **116113** | | **yes** |
| HNRNPA1P10 | heterogeneous nuclear ribonucleoprotein A1 pseudogene 10 | 664709 | | |
| MROH1 | maestro heat like repeat family member 1 | 727957 | | |
| HNRNPA1P16 | heterogeneous nuclear ribonucleoprotein A1 pseudogene 16 | 440396 | | |
| EFTUD1P1 | elongation factor Tu GTP binding domain containing 1 pseudogene 1 | 648809 | | |
| MUC5AC | mucin 5AC, oligomeric mucus/gel-forming | 4586 | | |
| POLR3H | polymerase (RNA) III subunit H | 171568 | | |
| PSMA2 | proteasome subunit alpha 2 | 5683 | | |
| EPT1 | ethanolaminephosphotransferase 1 | 85465 | | |
| SRCIN1 | SRC kinase signaling inhibitor 1 | 80725 | | |
| SUDS3 | SDS3 homolog, SIN3A corepressor complex component | 64426 | | |
| TIMM23 | translocase of inner mitochondrial membrane 23 | 100287932 | | |
| TUBB4A | tubulin beta 4A class IVa | 10382 | | |

Table S5: Proviral genes identified by Z score analysis.
